# Transactions of the American Med. Association

**Published:** 1855-11

**Authors:** 


Art. VI.—*The Transactions of the American Medical Association.* Vol. VIII. Philadelphia: 1855. pp. 763. 8vo.

---

We have received the eighth volume of the Transactions of our great medical congress, which is nearly as large, and presents quite as respectable an appearance, as any of its predecessors. In the short time that has been afforded us for an examination of its varied contents, we have not been able to give it more than a very cursory examination, but the little we have had time to read has satisfied us that, as a book of reference, it is of great and permanent value. Its statistics and reports constitute a mass of scientific knowledge to which the practitioner, teacher, and author must make frequent reference.

The report of Dr. Reyburn on the Diseases of Missouri and Iowa, covers nearly two hundred and forty pages, and embraces a vast amount of useful information. The second report, by Dr. Sanford B. Hunt, on the "Hygrometrical State of the Atmosphere in various Localities, and its influence on Health," is also an elaborate and exceedingly valuable paper; and it is followed by the report of Dr. Frank. H. Hamilton, on "Deformities after Fractures," many of the facts and conclusions of which we have had occasion before to refer to. Buffalo has been a large contributor to the Transactions of the Association. Two of her physicians have won the prizes offered for the best essay—Dr. Dalton, and Dr. Flint—and now we have in this volume two most creditable reports by Professors in the same University. The next report is on the "Diet of the Sick," and when we state that it is from the pen of Dr. Charles Hooker, of New Haven, it will not be worth while to add, that it is one of the best reports in the volume—scholarly, scientific, and practical. A report, by Dr. Wm. H. Byford, of Evansville, on "Scrofula," will be read with interest, as will also the one by Dr. N. S. Davis on the "Means of Preserving Milk, and on the Influence of Pregnancy and Menstruation on the composition and nutritive qualities of that Fluid." Reports on "Dysentery," by Drs. H. Taylor, and J. H. Beech, and on the "Effects of Alcoholic Liquors in Health and Disease," by our distinguished friend, Dr. Mussey, follow, and of the latter we need hardly say that it is a report worthy of the principles and practice of a veteran in the temperance cause. We trust that its venerable author may live long to write many more reports, and to illustrate the virtues of a system which he has faithfully pursued. The "Prize Essay on Placenta Prævia," is a paper of reference which none who are curious about the subject will be willing to dispense with.

We close this hurried, imperfect notice, with the account of the reception of the Association at "Independence Hall":—

"At twelve o'clock, M., May 2, the members of the American Medical Association, comprising above five hundred gentlemen, passed in procession through the Independence Square, and entered Independence Hall. A number of ladies were already present and the Hall was soon crowded to its utmost extent.

"Dr. Isaac Hays, of Philadelphia, in behalf of the Medical Association, introduced the members in the following address:—

"ADDRESS OF DR. ISAAC HAYS.

"*Mr. Mayor:*—I have the gratification of introducing to your Honor the Members of the American Medical Association—our National Medical Congress. This Association was organized, in our city, eight years ago, and has met annually since in one of the principal cities of the Union. It is composed of delegates from the Medical Societies and Colleges, Hospitals, and other Medical Institutions throughout our country. It was instituted with no selfish views, but to accomplish objects of the greatest importance to the public at large; to improve the healing art, and increase its powers for alleviating human suffering. It strives to attain these ends by securing more complete and thorough courses of instruction to students, and by raising the standard of requirements of those admitted into the ranks of the profession; by investigating the causes of the diseases which prevail in certain localities, and by seeking the way by which these causes may be removed, or their effects counteracted; by collecting reliable histories of the different epidemics, which from time to time spread over our country, and by endeavoring to discover the means of arresting their progress; by offering prizes for the most useful discoveries and improvements in medicine; and by promoting every measure tending to enlarge the boundaries of our science or increase the efficiency, and augment the usefulness of our art.

"In pursuance of these objects the Association has assembled this year in our city, and we feel deeply grateful to you for your courtesy in inviting us to visit this place, so venerated by every American heart.

"The philanthropy of our profession is not restricted to the cure of disease—it has a larger range; it embraces within its scope whatever tends to the improvement of our mental or moral condition, or even to confer 'the greatest good upon the greatest number.' Hence the profession have never been indifferent to national objects; on the contrary, they flatter themselves that they have not been behind any other class of the community in patriotism, but that they have been always prompt to serve their country, not only in their professional capacity, but also by their council in the Cabinet, and even by taking up arms for defence in the field.

"I *need not* remind your honor that the most illustrious physician of his day, in this country, the late Dr. Rush, was a member of the Congress of '76, and affixed his name to the Declaration of Independence; or that General Joseph Warren, one of the most eminent physicians of Boston, contributed by his eloquence to rouse his townsmen to resist the oppressive acts of the mother country, and shed his life's blood on Bunker Hill in defence of liberty; or that his younger brother, Dr. John Warren, animated by the same spirit, volunteered as a private soldier; nor need I refer you to other examples of a similar character. But I *may* assure you that the spirit which animated our ancestors, if it appear dormant, is not extinct in our bosoms, and that while standing on this spot—sacred to Human Liberty—we experience emotions kindred to those which the inspired Law-giver must have felt when he heard the voice calling to him out of the midst of the burning bush: 'Put off thy shoes from off thy feet, for the place whereon thou standest is holy ground.'"

---

### "REPLY OF MAYOR CONRAD.

"*Mr. Chairman of the Committee of Arrangements:*—I thank you in the name of the community which I have the honor to represent, for your eloquent introduction of our friends to the authorities of the city, and to this the Hall of Independence.

"*Gentlemen of the American Medical Association:*—I am proud of the privilege of extending to you, in the name of the government and of the people of Philadelphia, a most cordial welcome.

"I bid you welcome to our city—a city which, deriving a cherished distinction from the profession which you adorn, is eager, now and ever, to requite it, in her tribute of respect for its professors. I welcome you to our people, whose intercourse, for many a year, with you or your brethren, has inspired a feeling which, reserved as we are sometimes said to be, will, I doubt not, burst into earnest and unambiguous expression before you leave us.

"I welcome you, gentlemen, to this Hall, but not as strangers or the sons of strangers—for it is your own. As the temple and territory of Delphos, in the wildest domestic perturbations of Greece, afforded one sacred area over which the cloud of discord never gathered, one altar whose worship was never invaded, this spot, consecrated to our common American glory, knows no lines of latitude, and belongs, in truth, no more to us, whose peculiar privilege it is to inherit its guardianship, than to our brothers—*to you.* In coming hither, therefore, you *come home.* These precincts have been hallowed, for all time, by the heroic virtues of your and our fathers. This is the fountain from the which the living waters of American liberty were first drawn, and it is therefore most sacred—(woe to the generation in which it ceases to be sacred!)—but, like the well of the Patriarch, all the tribes of Liberty's Israel own here an equal right, and owe here an equal homage.

"In no sense, then, can I greet you as strangers—for yours are names familiar to every American proud of the science of his country; and those who are united, by this association, in a cause so lofty as that eloquently characterized by your Chairman, may not only claim the universal and acknowledged privileges of the Republic of minds, but the rights of a nearer and a dearer charter, the Brotherhood of beneficence—the kindred claims of noble hearts, knit in the highest and holiest of human aspirations. In this spirit, with the most fervent and fraternal sentiments of respect and regard, I greet and welcome you.

"You are right, Mr. Chairman, in claiming, amid the associations which hallow these precincts, a peculiar privilege for your profession—a profession which not only sprinkled, with the earliest sacrificial blood of the Revolution, the highest altar upon which Valor vowed and dedicated our country to freedom—I refer as you have referred, to Dr. Warren and Bunker Hill—but which in every struggle for the enlargement and enlightenment of human destinies, has been eminently distinguished for courage, zeal, and fidelity to the rights of man. You have, therefore, a peculiar right to claim kindred here, and have that claim allowed; and within these walls, which witnessed the zeal of Rush, it would be a treason to virtue to forget, that one of the lights of your profession shed glory upon the solemn debates of this hall, and was foremost among those that bade yonder bell* (preserved and devoted to the veneration of posterity), with its iron tongue, to PROCLAIM LIBERTY THROUGHOUT ALL THE LAND, TO ALL THE INHABITANTS THEREOF.

"It is the glorious peculiarity of your profession that, while Ambition, in its ordinary and most applauded paths, plays the part of the *destroyer*, and wins glory at the expense of human life and happiness, you and yours, with a more exalted civilization, a nobler heroism, have ever *sought to save.* Next to the highest of all human courage—if, indeed, it be merely *human*—that of the martyrs of religious truth—the courage of the physician, whether on the battle-field or in the lazar-house, the courage of science and humanity, is most sublime, and best entitled to the *clarum et venerabile nomen.* The vulgar courage of the warrior, under the base stimulus of passion, or the low greed of applause, can hardly be compared to the noble intrepidity of the surgeon, who gleans, in the ruthless and red-handed reaper's path, the leavings of the battle; and still less with the hero of the hospital, who encounters the grim antagonist in the horrid silence and gloom of the pestilence. Imagination can hardly embody an instance of human courage and virtue more sublime and unearthly than that of the physician, who, in the midnight of a plague-stricken city, thrids the fetid solitudes of its alleys, and, entering the devoted hovel of the wretched, ministers—while only Pestilence and Misery, Death and God look on—to the perishing. I need not step from this spot to grasp the hand of many a hero who claims no laurel—many a noble philanthropist whose sacred labors, in scenes like these, have been unmarked, save by the Eye that never slumbers, and remembered only by him who alone can reward.

"To such a profession, one venerable from its antiquity, noble from the grandeur of its objects, illustrious from its achievements, and which demands every aid and energy of genius and science, of head and heart, that dignifies the race, it is not strange that, go where it may, a ready homage meets and a ready blessing attends it. In our own city, all that is noble in patriotism, all that is exalted in science, all that is bright and beautiful in the arts which refine society, all that is lovely and cherished and holy in private life, combine to render the profession sacred and dear to us.

"There are few living to whom some one death in the past is

---

* *The Liberty Bell.*—This is the bell which was rejoicingly rung, from the steeple of the old State House, when the Declaration of Independence was originally read, in July, 1776, to the thousands assembled in the State House yard, now Independence Square. Upon this bell—cast long before the Revolution, and brought from England in the colony times—are the prophetic words of Scripture quoted: "Proclaim liberty throughout all the land, to all the inhabitants thereof."

not the sole event and solitary memory of the survivor's life—to him a lonely pyramid in the melancholy desert; and to such a mind and memory, *the debt of the death bed*, where science, rendered holy by its office, ministered, though never paid, is never repudiated. I never knew a good man, still less a good woman, who had not such a debt—a debt which bankrupt gratitude cherished with its holiest affections and sanctified with its devoutest memories.

"In these times, when the omnipotence of associated effort is invoked for so much that is of dubious merit, it is a gratifying spectacle to behold the enlightened professors of the most exalted of all arts—men sage and grave, unselfish and unaspiring—forsaking the homes to which they are bound by the affections and the afflictions of thousands, by wealth, and fame, and influence, to wander, wearily, away upon a pilgrimage of hundreds of leagues, in the cause and interests of the human family, its security, health and happiness For more than ten years, the representatives of your profession have thus gathered in Convention. What other body of our citizens have made a like effort—a like sacrifice? Selected from the most eminent of the profession, the delegates have been men whose years, like their virtues, were many. How difficult must have been, to them, the effort to burst through the bonds of a relying and clinching practice! How great the labor, and how heavy the sacrifice! They have already visited, in this duty, the cities of every section of our wide country. How many have fallen by the way-side? How many martyrs could you not thus number in this cause? How many of the good and great of the profession have, in these benevolent pilgrimages, joined the ranks of the thousands who have sacrificed themselves at the requisitions of duty, as recognized and enforced by your self-imposed laws—joining the dead in the effort to aid the living? The epitaph of the Spartans at Thermopylæ might well commemorate the virtues and the fate of these martyrs. But if the cost has been great, the results have been commensurate.

"Of the professional advantages attained, though I know them to be invaluable, I will not presume to speak; but I may be permitted to state, as health is the most important subject of municipal provision and care, that the *Transactions* of the Association, which I have examined with great interest, comprise much that merits the attention, and will reward the respectful consideration of the municipal governments of the Union.

"It is natural that Philadelphia should feel, as she does feel, a profound interest in the cause of medical education in this country. She cannot, of course, forget that it was here that the first medical college was established in this country; that its merits and success extorted a reluctant transatlantic tribute of admiration: and that, progressing rapidly, but wisely, it achieved and maintained an equality with the most celebrated institutions of the Old World. As the cause of medical education has expanded, and institutions worthy of the cause and the country have sprung up, each triumph, thus attained, has been regarded here as the successful outbursting of an offshoot from the primary effort; and Philadelphia, while rejoicing in the expansion and elevation of medical education throughout the land, has almost fancied—so earnest is her interest in medical education—that she had a right to indulge a *parental* pride in all that advances that interest.

"These genial feelings have been maintained, in all their early and fervid freshness, by constant intercourse with all sections of our country. The ingenuous and gallant youths that have come hither for medical instruction have, in their unstudied intercourse, exhibted the character of their respective States in a light so generous and exalted as to win our affections, not only for themselves, but for the communities and States which could exult in them as their own. Winter after winter, we have had many hundreds of these noble young spirits among us. And let me remark that, rigorous as I am said to be in the administration of the law, I have yet to know the first occasion to rebuke, much less to punish, a medical student. We have found them as gentle and decorous in their deportment as they are exalted in their aspirations; and had Philadelphia—eminently catholic in her affection for her sister communities—needed a lesson of love and loyalty, these high-hearted missionaries would have taught it. This interchange of sympathies has endured for the third of a century—may it last forever! The youths—youths no longer—who formerly bore those sentiments to the remote sections of our Republic, stand before me now as the reverend sages and ornaments of their profession, meeting here the evidences of a reputation which had preceded them, and has long been cherished by us. And who can tell what have been the results of this kindly interchange of kindly feelings? It has doubtless been felt in every commercial, social, and political relation of life, correcting the prejudices, harmonizing the discords, and subduing the dangers of our common country.

"We realize these facts. We recognize, in the members of an enlightened profession like yours, so many patriots and philanthropists, engaged in the great and general interests of the human race; and, apart from the mere scientific acquisitions of your annual meetings, we perceive in them results auspicious to all that we cherish, all that is kindly, forbearing, and conservative, between man and man, party and party, State and State, section and section; and, so regarding them, we hail and greet you with a welcome as sincere and cordial as the heart can conceive or the tongue can utter."

